# Study on the Complexation and Release Mechanism of Methylphenidate Hydrochloride Ion Exchange Resin Complex

**DOI:** 10.3390/polym13244394

**Published:** 2021-12-15

**Authors:** Conghui Li, Xiaolu Han, Xiaoxuan Hong, Xianfu Li, Hui Zhang, Zengming Wang, Aiping Zheng

**Affiliations:** State Key Laboratory of Toxicology and Medical Countermeasures, Beijing Institute of Pharmacology and Toxicology, 27th Taiping Road, Beijing 100850, China; angelina52819@163.com (C.L.); hanxiaolu921007@163.com (X.H.); hongxiaoxuan1216@163.com (X.H.); xiaofu0924@163.com (X.L.); zhhui58@126.com (H.Z.)

**Keywords:** ion exchange resin, molecular dynamics simulations, mechanism of complexation, drug release, methylphenidate hydrochloride

## Abstract

Since the advent of ion exchange resin, it has been widely used in many fields, including drug delivery systems. The drug binds to the resin through an exchange reaction to form a drug–resin complex, which can gradually release drugs through the exchange of physiological ions in the gastrointestinal tract, to realize functions such as taste masking and regulating release. In this study, the complexes of methylphenidate hydrochloride and Amberlite IRP69 were prepared and evaluated to explore the mechanism of complexation, influencing factors and release mechanism at a molecular level. Firstly, with the properties of the selected complexes, molecular dynamics simulation was innovatively used to find that the intermolecular interaction between drug molecules and ion exchange resin molecules is mainly caused by the stacking effect of π and salt bridges. Secondly, with the drug loading status as an indicator, the factors affecting the compounding process of the drug and resin were explored. Finally, the release mechanism of the drug–resin complex was studied by mathematical model fitting. In summary, a variety of methods were used to study the mechanism of complexation and release between drug and resin, providing a theoretical basis for promoting the marketing of ion−exchange resin−mediated oral preparations.

## 1. Introduction

**Figure 1:**
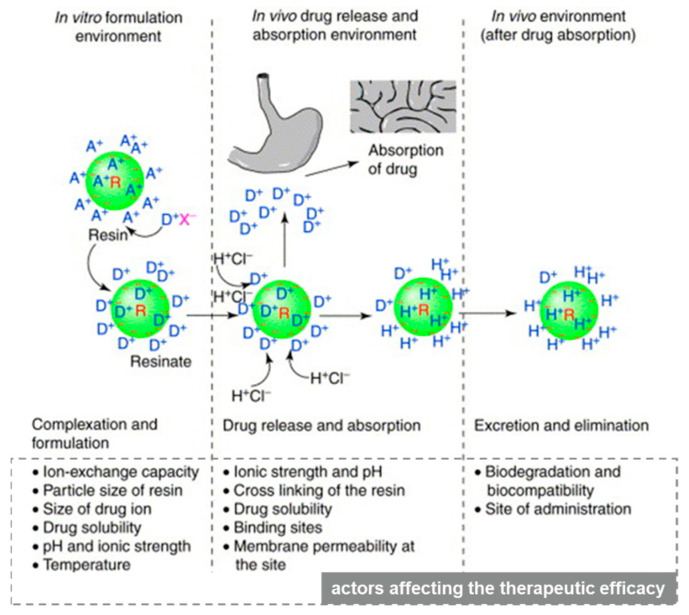
The basis of the ion−exchange process in drug delivery with factors affecting the therapeutic efficacy of the system at each stage. Reprinted by permission from Elsevier Publishers Ltd.: Drug Discovery Today. 2001, 6(17): 905–914, copyright 2021.

Ion exchange resin (IER) is a polymer that contains active groups in the molecule and can perform ion exchange with other substances [1]. In the solution, it can exchange its own ions with like−charged ions of the solution, which is a reversible chemical reaction. The molecular structure of ion exchange resin consists of three parts: [2] (1) net−like skeleton with three−dimensional structure; (2) immobile active group covalently connected to the net−like skeleton, also known as the functional group; (3) the active ion bonded to the active group by ionic bond having the opposite charge to the active group, also known as the counter ion. For example, in the structure of a sodium polystyrene sulfonate resin, the net−like skeleton is a polystyrene polymer, the active group is a sulfonic acid group, and the counter ion is a sodium ion [3]. The drug binds to the ion exchange resin through an exchange reaction to form a drug–resin complex, which can reversibly exchange sodium, potassium, or chlorine ions in the physiological environment [4]. In the drug release process of the drug–resin complex, cation exchange and anion exchange can be described by Equations (1) and (2), where A and B are the counter ions in the physiological environment. Figure 1 describes the factors that affect the ion exchange process involved in cationic drug delivery.

Resin^−^—Drug^+^ + A^+^ → Resin^−^—A^+^ + Drug^+^, (1)

Resin^+^—Drug^−^ + B^−^ → Resin^+^—B^−^ + Drug^−^, (2)

R and a green circle depict resin; a ‘−’ sign depicts the integral ion of the resin, and A^+^ is the counter ion. D^+^ is the drug ion, X^−^ is the ion associated with drug cation and H^+^CI^−^ is hydrochloric acid. Ions inside and outside the resin indicate ions adsorbed at the surface, as well as at the interior, of the resin structure [5].

Since the advent of IER in the middle of the 20th century, it has mainly been used in the fields of organic analytical chemistry and agriculture [5]. Until 1950, the application of IER in the pharmaceutical and biomedical fields was studied. Since then, IER has been extensively explored in drug delivery systems [6,7,8,9]. A literature report shows that IER is suitable for various drug delivery technologies, involving controlled release, rapid dissolution, site−specific, transdermal, iontophoretically assisted transdermal, nasal, topical, and taste−masking systems [5]. However, at present, there are few ion−exchange resin−mediated preparations on the market, and the vast majority of them use IER for taste masking for oral administration [10,11]. In the market, IER has almost no other role or use in other dosage forms. This is because the research on the mechanism of complexation between the drug and IER is still not comprehensive. The relevant literature in the past five years have introduced the preparation and characterization methods of the drug−ion exchange resin complex, and proved the control release of drug–resin complex by in vitro methods, and evaluated the taste−masking efficiency by integrated score evaluation method (ISEM) [12,13,14,15,16,17]. Most research on IER is still at the macro level and the stage of experience accumulation.

So far, most of the research on the mechanism of complexation stays at the macro level, rather than at the molecular level, which is still not comprehensive and in−depth. In this study, with methylphenidate hydrochloride (MPH) as a modal drug, molecular dynamics simulation was used to study the complexation mechanism of methylphenidate hydrochloride and ion exchange resin at the molecular level. At the same time, the physicochemical properties of the drug–resin complex, the release mechanism and the factors affecting the compounding process of the drug and the ion exchange resin were also studied. This study aims to provide a theoretical basis for promoting the marketing of ion−exchange resin−mediated preparations.

## 2. Materials and Methods

### 2.1. Materials

Pharmaceutical grade Amberlite™ IRP69 (IRP69), namely sodium polystyrene sulfonate (complex polymer), was obtained from Dow France S.A.S (Chauny, France) which is a strongly cationic IER with a sulfonic acid group (irregular particle). Methylphenidate hydrochloride (C_14_H_19_NO_2_·HCl, rod−shaped crystal) is dedicated from Institute of Pharmacology and Toxicology, Beijing. The reagents involved in this article, such as potassium chloride (KCl), sodium chloride (NaCl), dilute hydrochloric acid (HCl), and potassium dihydrogen phosphate (KH_2_PO_3_), are all analytical reagents (Sinopharm Chemical Reagent Co., Ltd., Shanghai, China).

### 2.2. Methods

#### 2.2.1. Preparation of Drug–Resin Complex

The drug–resin complex was prepared using the batch method [18,19]. Methylphenidate hydrochloride was dissolved in the deionized water, and then Amberlite™ IRP69 was added while the suspension was stirred continuously until the concentration of methylphenidate hydrochloride reached equilibrium. The concentration was detected by HPLC, referring to the assay method of methylphenidate hydrochloride sustained−release tablets in USP [20]. The drug–resin complex was obtained from the residue after centrifuging at 4000 rpm for 20 min, washed with the deionized water and then dried in an oven at 60 °C for 24 h.

#### 2.2.2. Scanning Electron Microscopy (SEM)

The surface morphology of IRP69, a physical mixture of methylphenidate hydrochloride and IRP69, methylphenidate hydrochloride and drug–resin complex were examined with SEM (JSM−7900F, Nippon Electronics Co., Ltd., Tokyo, Japan). Briefly, the samples were placed on double−sided carbon tape, coated with Pd and then observed under an operation voltage of 30 kV.

#### 2.2.3. Differential Scanning Calorimetry (DSC)

The thermal analyses of different samples were studied with a DSC instrument (LLC, TA instruments, Lukens, DE, USA). Briefly, 3 mg of each sample was placed on aluminum pans and then heated at a scanning rate of 5 °C/min from 20 to 350 °C. All the DSC examinations were conducted under a nitrogen atmosphere.

#### 2.2.4. Powder X−ray Diffraction (XRD)

The XRD measurements of these samples were carried out using a powder X−ray diffractometer (XPert3, PANalytical B.V., Eindhoven, The Netherlands) at 15 mA and 30 KV, the 2θ ranged from 3° to 50° with 0.02° step width and 5°/min scan speed.

#### 2.2.5. Fourier Transform Infrared (FT−IR) Spectrometry 

A FT−IR (Nicolet iS50; Thermo Fisher Scientific, Waltham, MA, USA) was used at the wavelength of 400 to 4000 cm^−1^, materials were pressed into sheets with KBr as the matrix [21].

#### 2.2.6. Molecular Dynamics Simulations

Two structures of ion exchange resin and methylphenidate hydrochloride molecule were constructed first using Gaussview 5 software (version: 5.0.8, Gaussian, Inc., Wallingford, CT, USA). The semi−empirical PM3 method was chosen to optimize the structure of ion exchange resin molecule because of its large systems, while the HF/6−31G* method was used to optimize the structure of methylphenidate hydrochloride molecules. After getting a stable configuration, 50 resin molecules were constructed and then molecular dynamics simulation for these 50 resin molecules was performed by using the Packmol program [22] (version: 18.16, University of Campinas, São Paulo, Brazil). After the system was stable, a certain concentration of methylphenidate hydrochloride molecules was added, and then the Packmol program was used to construct the initial mixture system and perform molecular dynamics simulation again. The HF/6−31G* and HF/STO−3G methods were separately used to fit the RESP [23] charge parameters of drug molecules and resins based on the antechamber program, and then the GAFF force field [24] parameters were constructed. Using the TIP3P water box model, the side length of the water box was set to 1 nm. Firstly, perform 5000 steps of energy minimization, then perform short−term 100 ps simulation under NVT and NPT ensemble, respectively, and finally generate 20 ns balance simulation. A cutoff radius of 1.0 nm, a time step of 2 fs, temperature of 300 K and pressure of 1 bar were used. The data were saved every 10 ps for later analysis. All ab initio calculations were carried out using the Gaussian09 program (version: 09, Gaussian, Inc., Wallingford, CT, USA), and all molecular dynamics simulations were performed on the Gromacs2018 program [25] (version: 2018, University of Groningen, Groningen, The Netherlands).

#### 2.2.7. Study of Factors Affecting the Compounding Process

The study was conducted with four independent variables, namely ratio of drug to IRP69, particle size of resin, temperature and type and strength of counter ions in system. The formulation and process with respective variables are as shown in Table 1. The results of this study will provide guidance on the optimal factors for complexation between methylphenidate hydrochloride and IRP69. Data are expressed as the mean ± SD.

#### 2.2.8. Drug Release Studies

Drug release studies (*n* = 3) of the drug–resin complex were conducted with paddle method at 75 rpm in different media with 900 mL to simulate drug release in physiological conditions in different parts of the digestive tract. The dissolution media were as follows: 0.4 M KH_2_PO_4_, phosphate buffer (pH 4.5), acetate buffer (pH 4.5), hydrochloric acid (pH 1.0), or distilled water, respectively. Samples were taken at pre−set time intervals and filtered for HPLC (Thermo, Waltham, MA, USA) analysis [26]. Data are expressed as the mean ± SD.

## 3. Results and Discussions

### 3.1. Characteristics of Drug−Resin Complex

**Figure 2:**
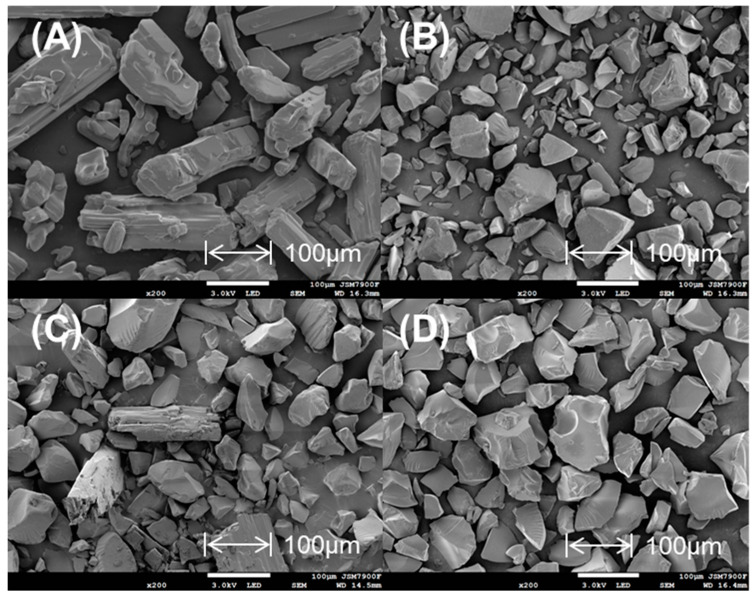
SEM images of (**A**) methylphenidate hydrochloride, (**B**) Amberlite IRP69, (**C**) a mixture of methylphenidate hydrochloride and Amberlite IRP69, (**D**) drug–resin complex.

As shown in Figure 2, SEM images of methylphenidate hydrochloride appeared as smooth rod−shaped crystals, while SEM images of Amberlite IRP69 demonstrated irregular and randomly shaped particles. In a mixture of methylphenidate hydrochloride and Amberlite IRP69, there still could be observed methylphenidate hydrochloride. However, the drug–resin complex particle characteristics appeared consistent with the resin, and it was difficult to find methylphenidate hydrochloride crystals in the drug–resin complex, which means that the combination of drug and resin is not a simple physical mixing.

**Figure 3:**
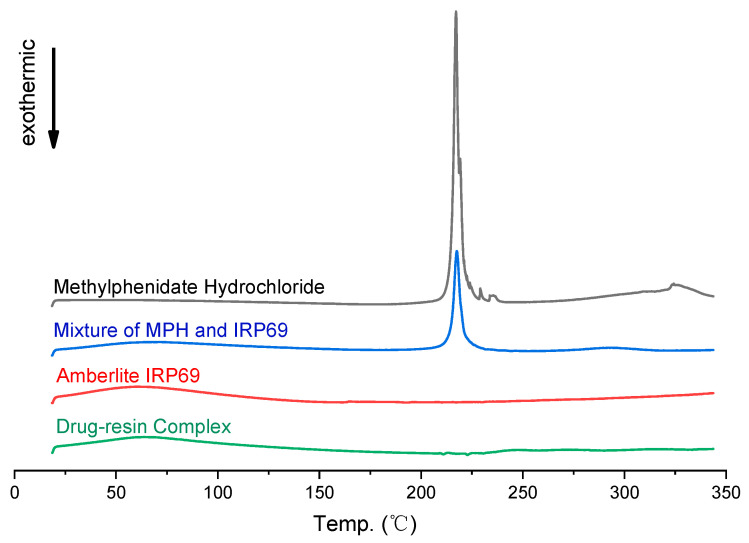
DSC patterns of methylphenidate hydrochloride, Amberlite IRP69, a mixture of methylphenidate hydrochloride and Amberlite IRP69, and drug–resin complex.

DSC patterns of methylphenidate hydrochloride exhibited endothermic peaks at around 225 °C (Figure 3), which was close to the intrinsic melting points. In the mixture of methylphenidate hydrochloride and Amberlite IRP69, there still could be found a similar sharp peak. Whereas the DSC curves of the drug–resin complex were similar to the ones of Amberlite IRP69, in which there was not observed a sharp peak. The peaks associated with methylphenidate hydrochloride did not appear in the drug–resin complex. The DSC patterns showed that the drug changed from the crystal structure to another form due to be loaded on the ion exchange resins.

**Figure 4:**
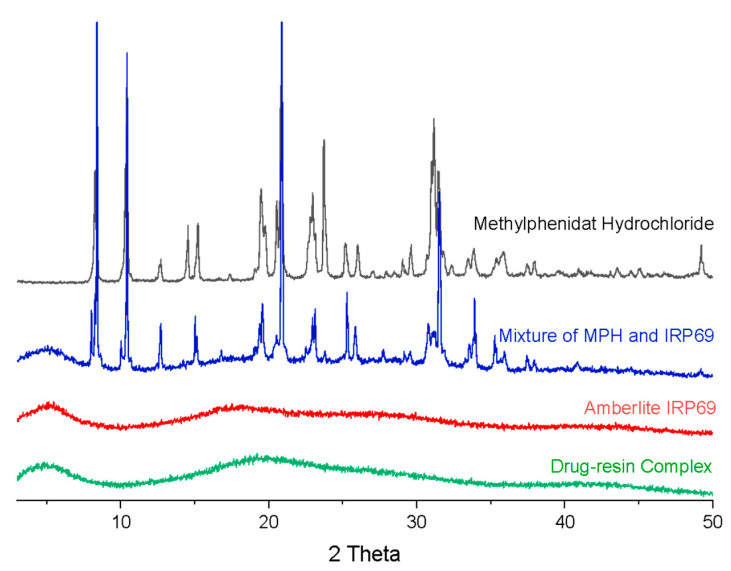
XRD patterns of methylphenidate hydrochloride, Amberlite IRP69, a mixture of methylphenidate hydrochloride and Amberlite IRP69, and drug–resin complex.

As shown in Figure 4., the XRD pattern of a mixture of methylphenidate hydrochloride and Amberlite IRP69 exhibited sharp peaks at 2θ of about 8.3°, 10.5°, 14.6°, 15.2°, 19.5°, 20.8°, 23°, 25.1°, 26° and 31.1°, in accord with the pattern of methylphenidate hydrochloride [27]. However, the drug–resin complex displayed no reflection peaks indicating the absence of crystalline methylphenidate hydrochloride. The results coincided exactly with DSC studies, reflecting that methylphenidate was mostly another form instead of a crystalline structure in the drug–resin complex systems.

The above research, in terms of morphology and crystal form, indicates the successful complexation of methylphenidate hydrochloride to resin, rather than simple physical mixing. Similar phenomena also occur in the compounding process of other drugs and ion exchange resins [18,28]. The results show that there is a certain interaction between the drug and the ion exchange resin, which significantly changes the original physicochemical properties of the drug.

### 3.2. Optical Properties of Drug−Resin Complex

**Figure 5:**
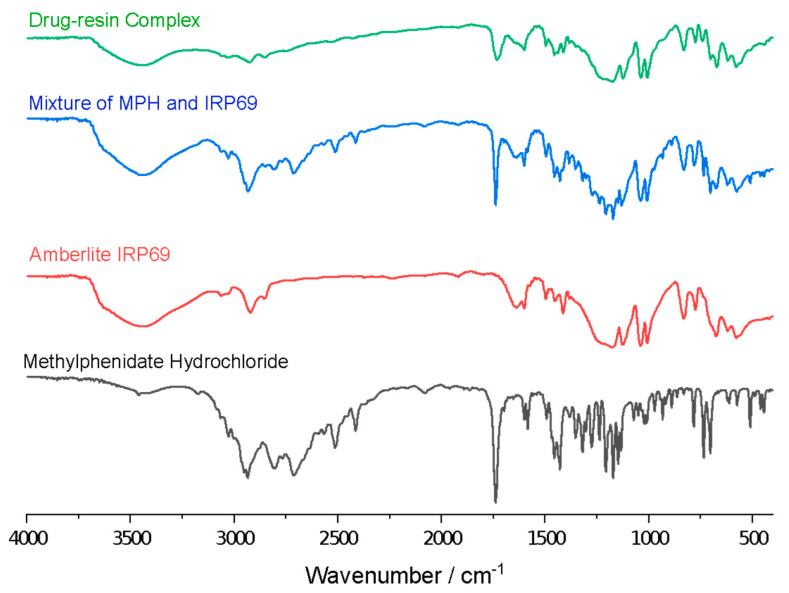
IR patterns of methylphenidate hydrochloride, Amberlite IRP69, a mixture of methylphenidate hydrochloride and Amberlite IRP69, and drug–resin complex.

FT−IR was used to analyze the structure and chemical bonds of methylphenidate hydrochloride, Amberlite IRP69, a mixture of methylphenidate hydrochloride and Amberlite IRP69 and drug–resin complex, so as to explore the interaction between methylphenidate hydrochloride and ion exchange resin. As shown in Figure 5, methylphenidate hydrochloride exhibited C−N stretching vibration in the range of 1250–1020 cm^−1^. In the spectra of the mixture of methylphenidate hydrochloride and Amberlite IRP69, these characteristic absorption bands did not show any significant change in the position. However, these peaks were greatly weakened and hardly observed in the drug–resin complex, indicating that there was appreciable interaction between the drug and ion exchange resin in the complex. This may be due to a restriction of the vibration associated with the increases of the steric hindrance and formation of hydrogen−bond during the complexation process [18,29]. These spectra images indicated the salt bridge formed between MPH’s NH groups and resin’s sulfonate group. The broadening and peak changes symbolize the formation of new chemical bonds [30,31]. In other studies on drug–resin complexes, the variation peaks of the complex is also considered to be the proof of the salt bridge [28].

### 3.3. Molecular Dynamics Simulations

The purpose of molecular simulation is to build a set of models and algorithms based on experiments and basic principles to calculate reasonable molecular structures and molecular behaviors. Not only can the static structure of the molecule be simulated, but the dynamic behavior of the molecular system can also be simulated [32]. In this simulation, the conformational stability and intermolecular interaction of the ion exchange resin were first investigated. Then the interaction between the drug molecule and the ion exchange resin was simulated. Finally, the changes in the forces of sodium ions and ion exchange resins after the addition of drug molecules were compared.

#### 3.3.1. Conformational Stability and Intermolecular Interaction of the Ion Exchange Resin

**Figure 6:**
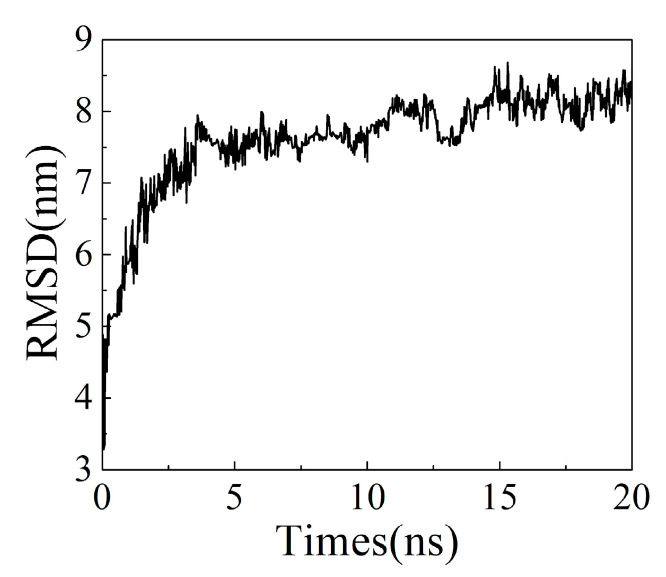
The root means square deviation (RMSD) of the structure vs. times.

**Figure 7:**
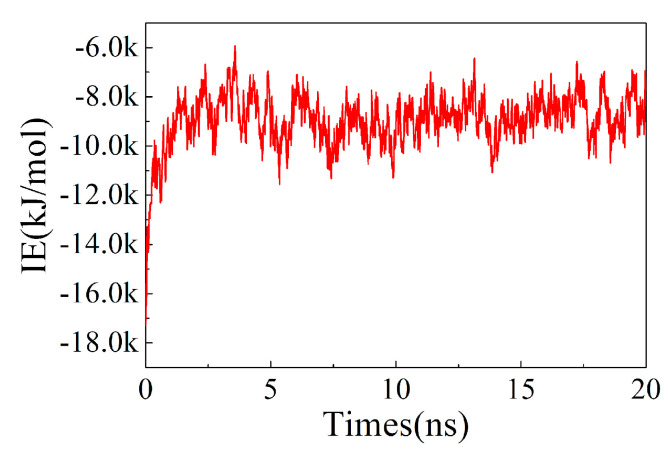
The interaction energy (IE) vs. times.

The analysis of the root mean square deviation (RMSD) of the structure can be used to investigate the conformational stability of ion exchange resins. As shown in Figure 6, the conformation of the ion exchange resin became stable after 5 ns, with its RMSD value was about 8 nm. Analyzing the intermolecular force of ion exchange resin showed that after 2.5 ns, the change of intermolecular interaction energy (IE) was stable and maintained between −8 k~−10 k kJ/mol (Figure 7).

#### 3.3.2. Interaction between Drugs and Ion Exchange Resins

**Figure 8:**
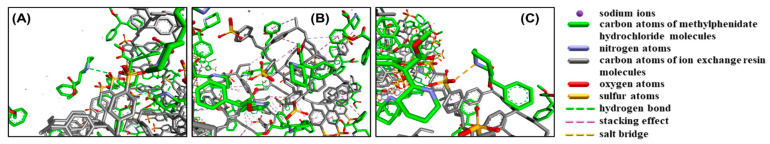
Simulation of the interaction between drugs and ion exchange resins.

When the system simulation became stable, methylphenidate hydrochloride molecules were added for investigation of their interaction with the ion exchange resin (Figure 8). First, there were hydrogen bonds between methylphenidate hydrochloride and ion exchange resin, which were mainly formed by the interaction between the nitrogen atom in the 6−membered ring hydrocarbon of the drug molecule and the S=O group in the resin structure. As shown in Figure 8A, the green dotted line indicates the hydrogen bond. Besides, there is a stacking effect between the drug and the ion exchange resin, represented by the pink dotted line in Figure 8B, including the CH_3_−π interaction formed by the methyl group of the drug molecule and the resin benzene ring, and π−π interaction of the benzene ring of the drug molecule and the resin. Most importantly, there is a salt bridge (represented by the orange dotted line in Figure 8C) between the nitrogen atom in the 6−membered ring hydrocarbon of the drug molecule and the S−O group in the resin structure, which is consistent with FT−IR study.

**Figure 9:**
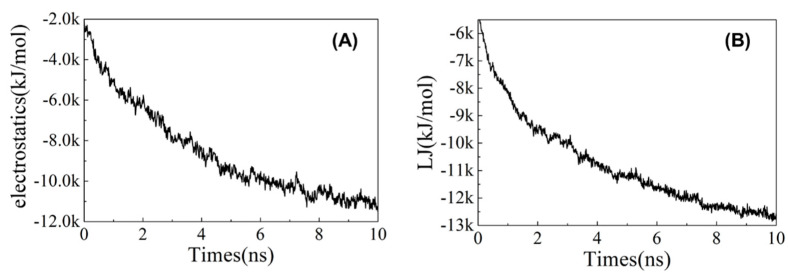
The (**A**) electrostatic energy and (**B**) LJ potential energy between drug molecules and ion exchange resin molecules vs. times.

Analyze the electrostatic energy and Lennard−Jones (LJ) potential energy between drug molecules and ion exchange resin molecules. As shown in Figure 9, the electrostatic energy was basically stable after 8 ns, fluctuating around −11,000 kJ/mol on average, while LJ potential energy tended to stabilize at −12,500 kJ/mol after 8 ns. The results indicate that the contribution of the electrostatic energy and LJ potential energy between drug molecules and ion exchange resin molecules is both very important, yet the contribution of the LJ potential energy is stronger.

**Figure 10:**
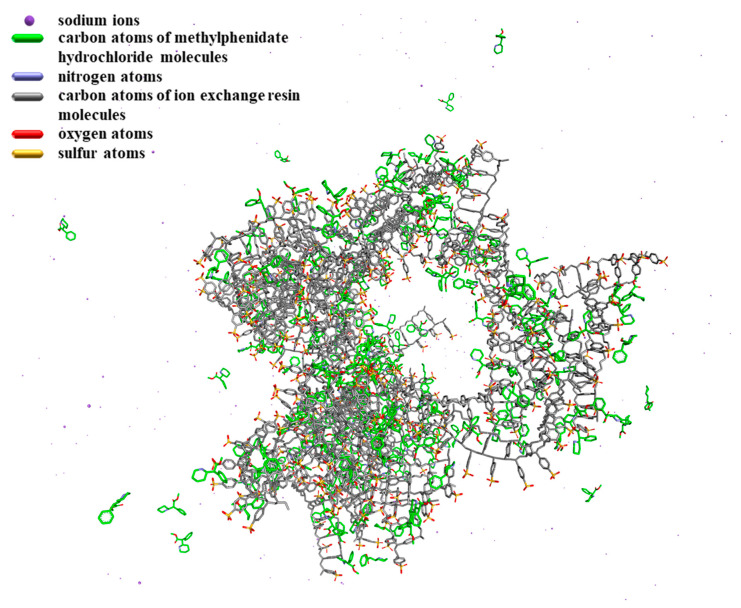
The conformation of the system of methylphenidate hydrochloride and ion exchange resins.

The conformational analysis of the system is shown in Figure 10. When drug molecules were added to the system, they formed hydrogen bonds, stacking effects and salt bridges with ion exchange resin molecules. According to the analysis of the electrostatic energy and LJ potential energy, it can be known that both the electrostatic energy and LJ potential energy make a great contribution to the intermolecular interaction. Therefore, the intermolecular interaction between drug molecules and ion exchange resin molecules is mainly caused by the stacking effect of π and salt bridges.

#### 3.3.3. Change of Interaction between Sodium Ions and Ion Exchange Resins

**Figure 11:**
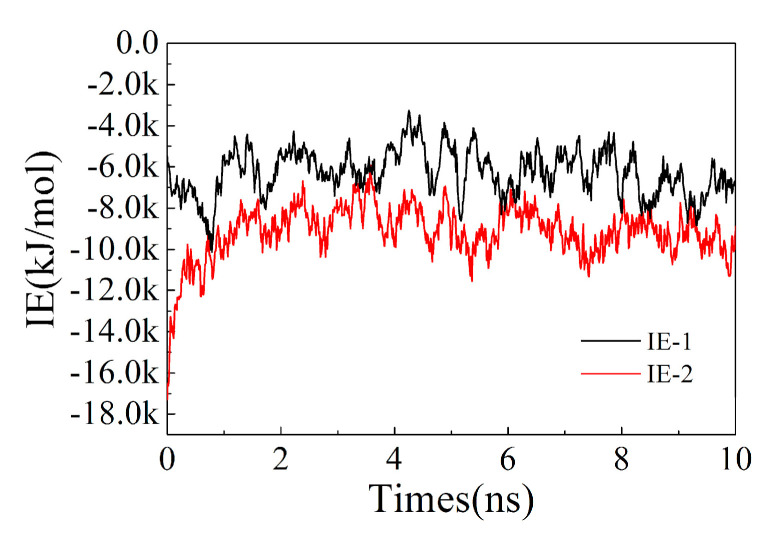
Change of interaction between sodium ions and ion exchange resins. IE−1 represents the interaction energy (IE) between sodium ions and ion exchange resins before the addition of methylphenidate hydrochloride molecules, while IE−2 represents the interaction energy after the addition of methylphenidate hydrochloride molecules.

Compare the changes of interaction energy between sodium ions and ion exchange resins before and after adding drug molecules in the system. In the whole process of molecular dynamics simulations, the statistics of the interaction energy between sodium ions and ion exchange resins are shown in Figure 11. After adding the drug molecules, the interaction energy between sodium ions and ion exchange resins significantly decreased. The result indicates that the addition of methylphenidate hydrochloride molecules reduces the interaction between sodium ions and ion exchange resin molecules and promotes the dissociation of sodium ions from the ion exchange resin.

### 3.4. Factors Affecting the Compounding Process

#### 3.4.1. Exchange Capacity of Ion Exchange Resin

Prepare drug–resin complex and determine the concentration of the remaining drug in the solutions, and calculate the exchange capacity, as depicted in the following Equation (3) [1,33]:

(3) $$X=\frac{\left(C_{0}-C\right)\times V}{M},$$

where X is the exchange capacity, $C_{0}$ is the initial concentration of drug in the solution, C is the concentration of drug in the solution at a certain moment, V is the volume of solution, and M is the mass of dry resin.

When the concentration of drug reaches equilibrium, the exchange capacity at equilibrium is calculated as depicted in the following Equation (4)

(4) $$X_{e}=\frac{\left(C_{0}-C_{e}\right)\times V}{M},$$

where $X_{e}$ is the exchange capacity at equilibrium, $C_{e}$ is the balanced concentration of the drug in the solution.

**Figure 12:**
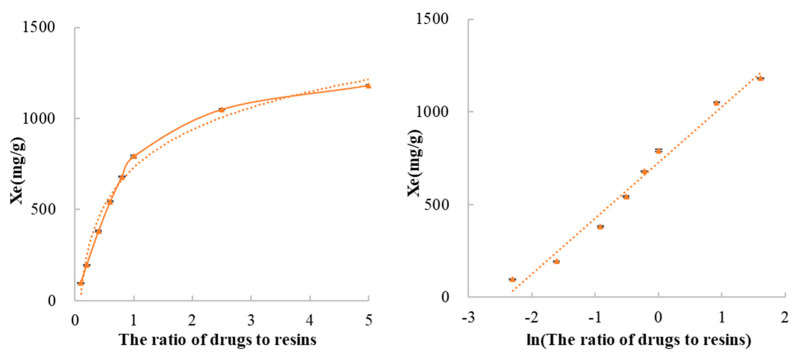
The effect of the ratio of drugs to resins on the exchange capacity at equilibrium.

The drug to resin complexes are, respectively, prepared at ratios of 1:10, 2:10, 4:10, 6:10, 8:10, 10:10, 10:4 and 10:2. In the case of different ratios of drugs to resins, when methylphenidate hydrochloride and Amberlite IRP69 complex systems reached equilibrium, the parameters such as drug concentration and exchange capacity were illustrated in Table 2 and Figure 12. As the ratio of drugs to resins increases, the exchange capacity at equilibrium (*X_e_*) has a significant upward trend. In this system, there is a high exchange capacity of ion exchange resin.

Analyzing the data of *X_e_* and the ratio of the ratio of drugs to resins by using nonlinear least−square theory, it was found that there existed a functional relation between the value of *X_e_* and the ratio of drugs to resins. The functional relationship is described as the following Equation (5), and the correlation coefficient (r) is 0.9905.

(5) $$X_{e}=301.08lnR+730.03,$$

where R is the ratio of drugs to resins.

In other words, there is a linear correlation between the exchange capacity at equilibrium and the natural logarithm value of the ratio of drugs to resins. This linear correlation helps to determine the ratio of drug to resin to prepare complexes with different drug loadings to meet different clinical requirements.

#### 3.4.2. Particle Size of Ion Exchange Resin

**Figure 13:**
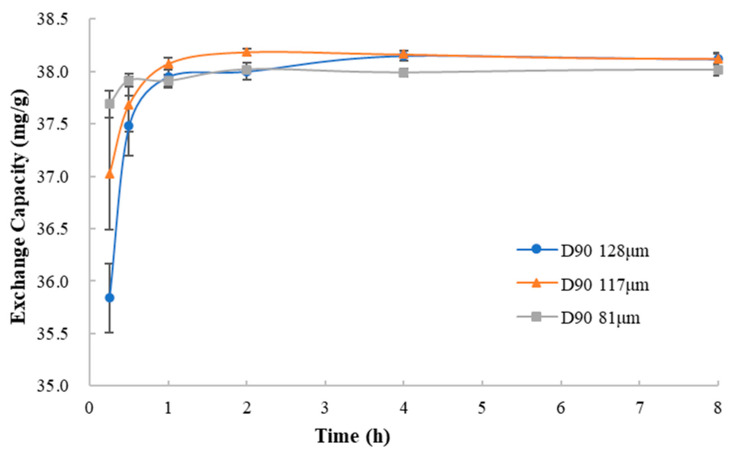
The curves of the exchange capacity vs. mixing time with the resins of different particle size.

To investigate the effect of resin particle size on the process of ion exchanging, ion exchange resins of different particle sizes were used to mix with methylphenidate hydrochloride in the deionized water under the same condition. Comparing the curves of the exchange capacity vs. mixing time (Figure 13), in the initial 0.25 h, the ion exchange rate (expressed as the ratio of exchange capacity and time) of ion exchange resins of different particle sizes was 143.35 mg/g/h (D90 = 128 μm), 148.08 mg/g/h (D90 = 117 μm) and 150.76 mg/g/h (D90 = 81 μm). It could be seen that the ion exchange rate of the resin with larger particle size was slightly slower than that of the resin with smaller particle size. When resin particles get larger, the penetration rate of drug solution into the resin particles and the rate of the ion exchange are slower. The results indicated that the gradual penetration of the drug solution into the resin particles for further contact was necessary for ion exchange between the drug and the resin.

#### 3.4.3. Time of Mixing

Observing the curves of the exchange capacity and mixing time, as shown in Figure 13, it is found that it takes a certain time for the drug to be loaded on the resins to achieve equilibrium. This time includes both the ion exchange process and the process where the drug solution completely penetrates the resin particles. Hence, within a certain range, mixing time can affect drug loading of resins. To prepare a drug–resin complex with appropriate drug loading, mixing time should be identified by experiments.

#### 3.4.4. Temperature of Mixing Complexation

**Figure 14:**
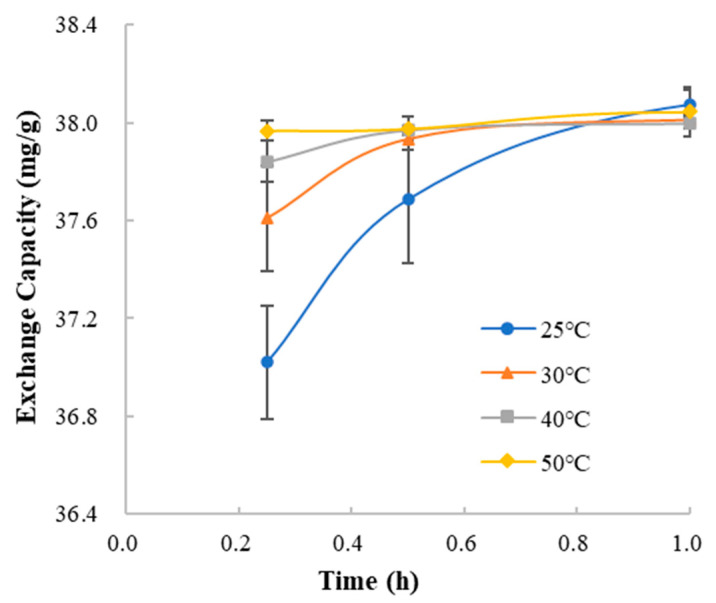
The curves of the exchange capacity vs. mixing time at different temperatures.

In the deionized water, compare the curves of the exchange capacity and mixing time at different temperatures (Figure 14). At each temperature, the ion exchange rate in the initial 0.25 h was 148.08 mg/g/h (25 °C), 150.43 mg/g/h (30 °C), 151.37 mg/g/h (40 °C) and 151.87 mg/g/h (50 °C). The result indicated that the temperature of mixing influenced the ion exchange between methylphenidate hydrochloride and Amberlite IRP69. Higher temperature led to faster ion exchange rate, which could be due to the faster molecular motion.

#### 3.4.5. Type and Strength of Counter Ions in System

The dissociation of the drug to form ions in the solution is the prerequisite for ion exchange between the drug and the resin. Therefore, in the process of ion exchange between methylphenidate hydrochloride and Amberlite IRP69, the presence of other counter ions in the solution can competitively inhibit the exchange of methylphenidate ions and sodium ions of Amberlite IRP69. In order to explore this competitive inhibition, the exchange capacity at equilibrium was investigated in an ion exchange system containing different types and concentrations of counter ions (1.25 mol/L and 2.5 mol/L of Na^+^, K^+^ and H^+^).

**Figure 15:**
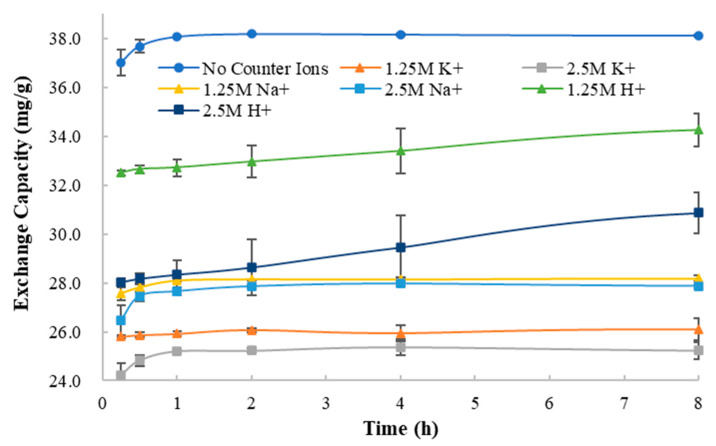
The curves of exchange capacity vs. mixing time with different types and concentrations of counter ions.

As shown in Figure 15, because of the presence of Na^+^, K^+^ and H^+^, the exchange capacity of the system decreased significantly. The degree of decrease caused by different counter ions is not the same. According to the results of the experiment, the presence of potassium ions causes the most significant reduction in exchange capacity, followed by sodium ions, and hydrogen ions have the least impact. 

As well, the higher the concentrations of counter ions are, the lower the exchange capacity of the system is. The extent of this effect is also related to the type of counter ion. In the drug–resin complex systems with H^+^, the concentration of counter ions had the greatest impact on exchange capacity, while in the systems with Na^+^, the concentration of counter ions had the least impact. The different influences of different counter ions on the exchange capacity are related to the interaction energy between the different counter ions and the ion exchange resin molecules.

Compared with the curves of exchange capacity and mixing time, it was found that the existence of H^+^ greatly prolongs the time for the ion exchange system to reach equilibrium. However, other counter ions had no such effect. The reason is that methylphenidate hydrochloride is a weakly acidic drug, and the hydrogen ions in the solution can inhibit the dissociation of methylphenidate hydrochloride, thereby reducing the rate of ion exchange between methylphenidate hydrochloride and the resin.

In summary, the existence of different types and concentrations of counter ions reduces the exchange capacity of the ion exchange systems to varying degrees, which means that the presence of the counter ions competitively inhibits the ion exchange process of methylphenidate hydrochloride and ion exchange resins. At the same time, hydrogen ions reduce the ion exchange rate by inhibiting the dissociation of the drug, indicating that the dissociation of the drug is a necessary step for ion exchange with the resin. These results prove that there are two processes in the exchange process between the drug and the ion exchange resins: the dissociation of the drug and the ion exchange resin in the systems and the exchange between the drug cation and the resin cation.

### 3.5. Mechanism of Release

According to literature and previous research, drug releases from drug–resin complexes are composed of particle diffusion and film diffusion processes. Hence, the drug release kinetics of the drug–resin complexes mainly depend on the drug and counter ion diffusion resistance in the core of the particles and the boundary layer surrounding the particles [28,34]. The processes involved in the release phase of the drug–resin complexes include: (1) diffusion of counter ions in boundary layers and drug–resin complexes, (2) reaction of ion exchange between counter ions and drug, and (3) diffusion of the dissociated drug out of the resin through boundary layers. Compared with other processes, the ion exchange reaction between the counter ion and the drug is very fast and has little effect on the overall drug release kinetics [34].

Based on the exchange process as reversible, the impact of type and strength of counter ion in the system on drug–resin compounding will absolutely further impact the process of drug release from the complex. Therefore, it is necessary to reveal the release mechanism of the complex in different parts of the digestive tract by studying the release behavior of the complex in a variety of dissolution media. This may help to achieve controlled drug release and targeted drug delivery to decrease the adverse effects of drug [35]. It is of great significance for the development of ion exchange resin−mediated formulations.

**Figure 16:**
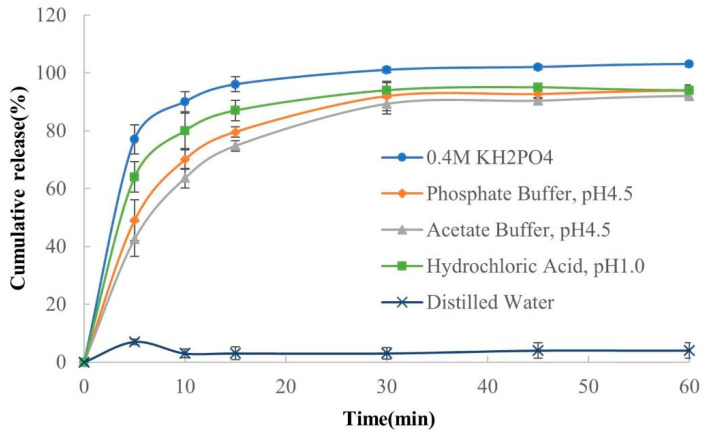
The release profiles of drug–resin complex in different dissolution media (*n* = 3, mean ± SD).

As shown in Figure 16, there are significant differences in the release profiles of the drug–resin complex in different dissolution media. It was observed that the release amount was the highest in 0.4 M KH_2_PO_4_ solution, while the release amount in water was almost zero. This is because the different amounts and types of counter ions in different dissolution media result in different amounts of ion exchange between counter ions and drug ions, leading to the different release amount of the drug from the complex. There are nearly no counter ions in distilled water, so the complexes are hardly released in it, which is also regarded as a tool to evaluate the reliability of taste masking [18,36].

**Figure 17:**
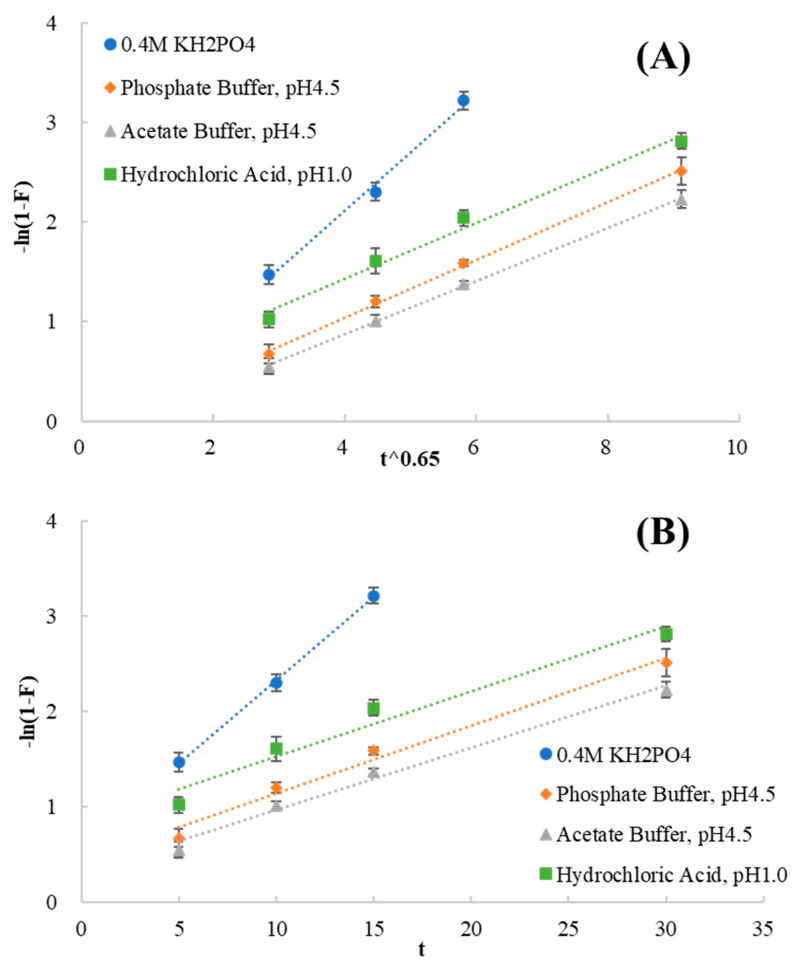
The release profiles from drug–resin complexes plugged into particle diffusion controlled model (**A**) and film diffusion controlled model (**B**).

According to Boyd’s and Bhaskar’s theory, the release profiles from drug–resin complexes were entered into the particle diffusion (Figure 17A) and film diffusion (Figure 17B) controlled models. Kinetic parameters for release data were analyzed as shown in Table 3. In the media of 0.4 M KH_2_PO_4_, the value of the correlation coefficient (r) obtained using the film diffusion controlled was higher than that of the particle diffusion controlled. In this case, the film diffusion controlled model was the rate−limiting step. In the other media, the release results better fit the particle diffusion controlled model with the higher linearity. The results indicated that the particle diffusion process was the rate−limiting step in the other media.

## 4. Conclusions

The mechanism of complexation between the drug and the ion exchange resin has been explored. This study confirms that the drug and the ion exchange resin form complexes through salt bridges, rather than simply mixing. The complex mainly relies on the stacking effect of π and salt bridges to maintain conformational stability. It has been revealed that the increase of temperature and the decrease of resin particle size have a positive effect on the compound of methylphenidate hydrochloride and Amberlite IRP69. Based on the ability to inhibit drug dissociation and the interaction energy between the different counter ions and the ion exchange resin molecules, the type and strength of the counter ion in the system have a competitive inhibitory effect on the compounding process. The particle diffusion controlled model was the rate−limiting step in most media, which can provide a reference for the choice of administration route and dosage form. This research can be used as a basis for predicting the compounding and release capabilities of drugs and resins, to help realize high−throughput screening of drugs and design prescriptions and processes. It is hoped that this can help to promote the application of ion exchange resins in the field of medicine preparations.

## Figures and Tables

**Table 1:** Study of factors affecting the compounding process.

No.	Factor	The Ratio of Drugs to Resins	Particle Size of Ion Exchange Resin (D90, μm)	Temperature (°C)	Type and Strength of Counter Ions in System
1	The ratio of drug to resin	1:10	117	25	No Counter Ions
2		2:10	117	25	No Counter Ions
3		4:10	117	25	No Counter Ions
4		6:10	117	25	No Counter Ions
5		8:10	117	25	No Counter Ions
6		10:10	117	25	No Counter Ions
7		10:4	117	25	No Counter Ions
8		10:2	117	25	No Counter Ions
9	Particle Size of Ion Exchange Resin	4:10	128	25	No Counter Ions
10		4:10	117	25	No Counter Ions
11		4:10	81	25	No Counter Ions
12	Temperature	4:10	117	25	No Counter Ions
13		4:10	117	30	No Counter Ions
14		4:10	117	40	No Counter Ions
15		4:10	117	50	No Counter Ions
16	Type and Strength of Counter Ions in System	4:10	117	25	No Counter Ions
17		4:10	117	25	1.25 M K+
18		4:10	117	25	2.5 M K+
19		4:10	117	25	1.25 M Na+
20		4:10	117	25	2.5 M Na+
21		4:10	117	25	1.25 M H+
22		4:10	117	25	2.5 M H+

**Table 2:** Methylphenidate hydrochloride concentration in the drug–resin complex systems.

The Ratio of Drugs to Resins	*C*_0_ (mg/mL)	*C_e_* (mg/mL)	*V* (mL)	*M* (g)	*X_e_* (mg/g)
1:10	40.00	0.274	100	40.005	99.30
2:10	40.00	0.614	100	20.003	196.90
4:10	40.00	1.80	100	10.000	381.97
6:10	60.09	5.66	100	10.006	543.95
8:10	80.01	11.97	100	10.000	680.40
10:10	40.00	8.31	100	4.002	791.94
10:4	40.00	23.20	100	1.600	1050.19
10:2	40.00	30.54	100	0.801	1181.36

**Table 3:** Kinetic parameters for release data.

Medium	Particle Diffusion Controlled Model		Film Diffusion Controlled Model	
	Equation	r	Equation	r
0.4 M KH_2_PO_4_	−ln(1 − F) = 0.5870 t^0.65^ − 0.2382	0.9967	−ln(1 − F) = 0.1749 t + 0.5812	0.9996
Phosphate Buffer, pH4.5	−ln(1 − F) = 0.2911 t^0.65^ − 0.1240	0.9993	−ln(1 − F) = 0.0713 t + 0.4251	0.9924
Acetate Buffer, pH4.5	−ln(1 − F) = 0.2662 t^0.65^ − 0.1891	0.9997	−ln(1 − F) = 0.0653 t + 0.3120	0.9940
Hydrochloric Acid, pH1.0	−ln(1 − F) = 0.2811 t^0.65^ + 0.3077	0.9933	−ln(1 − F) = 0.0684 t + 0.8453	0.9794
